# Barriers to the adoption of routine surgical video recording: a mixed-methods qualitative study of a real-world implementation of a video recording platform

**DOI:** 10.1007/s00464-024-11174-2

**Published:** 2024-08-15

**Authors:** Kyle Lam, Catherine Simister, Andrew Yiu, James M. Kinross

**Affiliations:** Department of Surgery and Cancer, Imperial College, 10th Floor Queen Elizabeth Queen Mother Building, St Mary’s Hospital, London, W2 1NY UK

**Keywords:** Surgical video recording, Surgical data science, Artificial intelligence, Digital surgery

## Abstract

**Background:**

Routine surgical video recording has multiple benefits. Video acts as an objective record of the operative record, allows video-based coaching and is integral to the development of digital technologies. Despite these benefits, adoption is not widespread. To date, only questionnaire studies have explored this failure in adoption. This study aims to determine the barriers and provide recommendations for the implementation of routine surgical video recording.

**Materials and methods:**

A pre- and post-pilot questionnaire surrounding a real-world implementation of a C-SATS^©^, an educational recording and surgical analytics platform, was conducted in a university teaching hospital trust. Usage metrics from the pilot study and descriptive analyses of questionnaire responses were used with the non-adoption, abandonment, scale-up, spread, sustainability (NASSS) framework to create topic guides for semi-structured interviews. Transcripts of interviews were evaluated in an inductive thematic analysis.

**Results:**

Engagement with the C-SATS^©^ platform failed to reach consistent levels with only 57 videos uploaded. Three attending surgeons, four surgical residents, one scrub nurse, three patients, one lawyer, and one industry representative were interviewed, all of which perceived value in recording. Barriers of ‘change,’ ‘resource,’ and ‘governance,’ were identified as the main themes. Resistance was centred on patient misinterpretation of videos. Participants believed availability of infrastructure would facilitate adoption but integration into surgical workflow is required. Regulatory uncertainty was centred around anonymity and data ownership.

**Conclusion:**

Barriers to the adoption of routine surgical video recording exist beyond technological barriers alone. Priorities for implementation include integration recording into the patient record, engaging all stakeholders to ensure buy-in, and formalising consent processes to establish patient trust.

**Supplementary Information:**

The online version contains supplementary material available at 10.1007/s00464-024-11174-2.

Minimally invasive techniques are standard practice for many surgical procedures performed worldwide [1]. However, despite this widespread use, only a minority of surgeons routinely record video for use beyond the perioperative period. A recent nationwide study demonstrated significant variations in the practices of surgical video recording in the UK, estimating that just one fifth of surgeons routinely record their procedures [2]. This is surprising as the benefits of routine video recording are multiple. Surgical video acts as an objective documentation of the operative record [3–5], is an invaluable resource for training [6, 7], and is a substantial data resource forming the foundation for many digital surgical applications [8]. Lack of data has been consistently cited as a significant barrier to progression of digital surgery [8–10] and the scale of surgical video datasets [9] are minute when compared to those within conventional computer vision research [11].

The failure to routinely record surgical video has often been attributed to a lack of available infrastructure [12]. However, a range of recording and surgical analytics products are now available on the market, including C-SATS^©^ (Johnson and Johnson) and TouchSurgery™ (Medtronic), suggesting that the reason for non-adoption extends beyond technological barriers alone. Some of these hypothesized wider barriers include challenges to privacy both for patients and surgeons [13, 14], concerns that recordings may result in an increase in medicolegal action against surgeons [5, 15], and fears that routine video recording may lead to a Hawthorne effect [16, 17].

To date, the current literature exploring these barriers have comprised of questionnaire studies which are restricted in their exploratory scope. This mixed-methods study aims to build on the existing literature by completing an in-depth qualitative assessment of the barriers to routine surgical video recording. We utilize usage data from a real-world surgical video recording platform pilot study and include participants to contextualize barriers in a real-world clinical setting. From these results, recommendations for wider implementation of routine surgical video recording are derived to facilitate other researchers and institutions.

## Methods

**Fig. 1:**
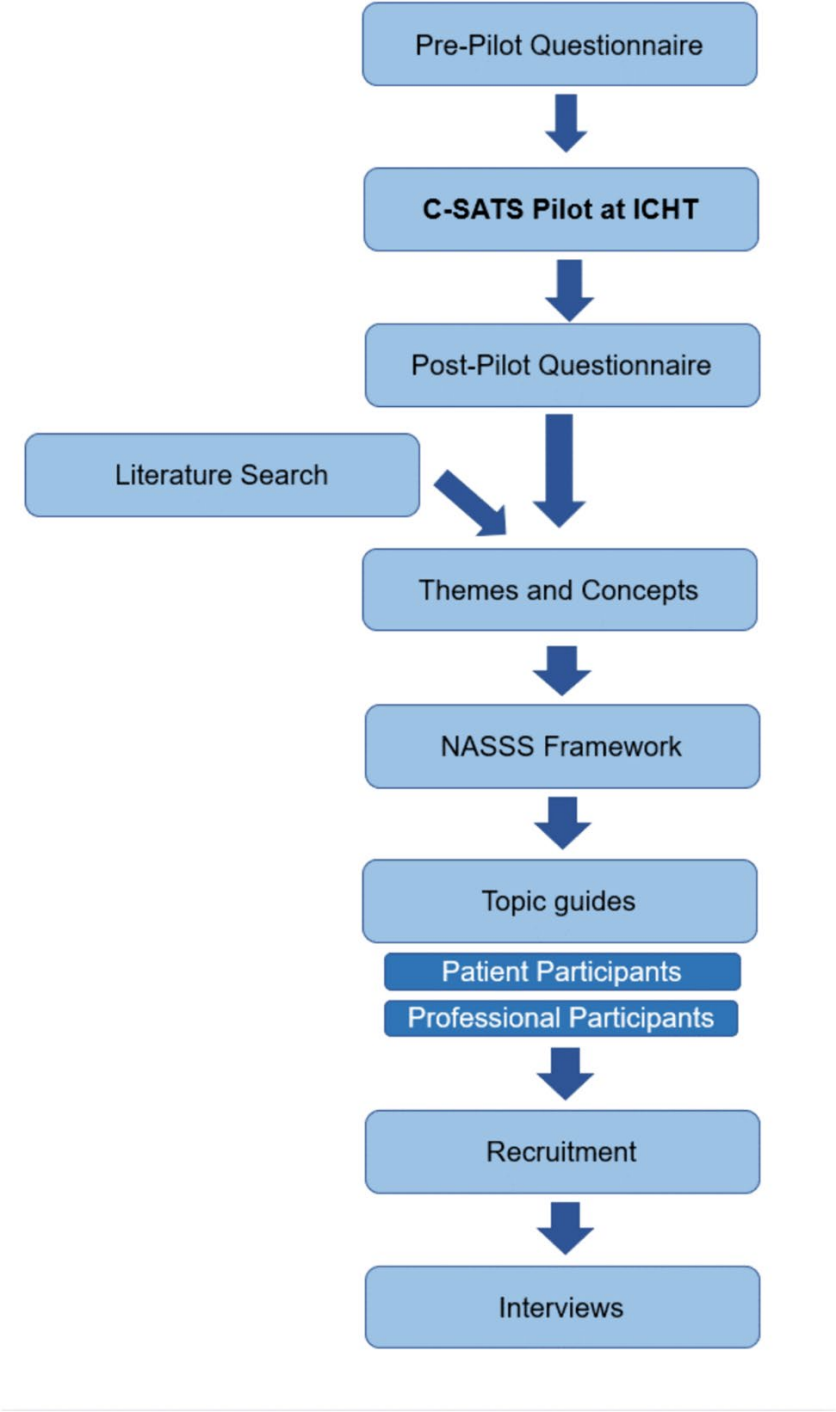
Flow diagram representing the relationship between the questionnaire in phase 1 of this study, and the semi-structured interviews in phase 2

This study consisted of two phases (Fig. 1). Phase 1 consisted of the deployment of C-SATS^©^ in a UK university teaching hospital trust. Phase 2 consisted of semi-structured interviews to explore perceptions, perceived utility, and barriers to implementation for routine surgical video recording.

### Phase 1: Deployment of a surgical video recording platform

#### C-SATS^©^ pilot

Phase 1 of this study incorporated a 44-week pilot of C-SATS^©^, a recording and surgical analytics platform at a UK university teaching hospital trust. The platform included a video library of expert cases as well as the function to record, review and receive feedback on individual procedures. All surgical residents and attendings across all specialties were invited to participate. The C-SATS^©^ device was installed into ten operating rooms across three hospitals for 31 weeks capturing the stream from the endoscopic camera once the device had been manually activated, and user details inputted. Users had access to a video library of expert cases and an AI-powered analytics dashboard for longitudinal performance tracking. Engagement with the platform was monitored throughout and interventions, such as email reminders, were utilized if engagement dropped at any point.

#### Analysis

Pre- and post-pilot questionnaires were distributed to participants (Appendix 1–3). Questions focussed on satisfaction with current feedback from training procedures, opinion of video-based review, and AI methodologies for training. Questionnaire responses were used to extract themes to design topic guides for semi-structured interviews. Metrics regarding engagement and video uploads to the C-SATS^©^ platform were also analyzed.

### Phase 2: Semi-structured interviews

#### Topic guides

Topic guides were created by two authors (KL and CS), with separate guides for professional and patient participants. Themes identified from the questionnaire and the current literature were used to inform the questions. Topic guides were structured around the NASSS framework, which is defined as ‘an overarching theory of complex adaptive systems’ [18] (Appendix 4–6). The framework considers multiple domains within a system, and further stratifies by the assumed difficulty when implementing in this area. Thus, it allows for the thorough identification and weighting of barriers highlighted. This framework was chosen due to the identification of the complexity in introducing routine recording in the literature.

#### Study participants

Members of the extended surgical team were recruited using snowball sampling. All surgeons participating in the C-SATS^©^ pilot were invited to participate, but pilot involvement was not a requirement. Industry, information governance, legal representatives, and patients were also invited to participate as key stakeholders. Patient participants were eligible if on a waiting list for surgery or had previously undergone surgery in the last 3 years.

#### Data collection

A grounded theory approach using a constructivist research paradigm was chosen to allow ideas to emerge from the data. Interview findings were reported using the Standards for Reporting Qualitative Research (SRQR) (Appendix 7). All interviews were conducted over Microsoft Teams (Microsoft, Redmond, WA, USA) or telephone call (CS). All interviews were recorded with informed consent. Transcripts were manually checked and anonymized for analysis.

#### Data analysis

An inductive thematic analysis was conducted (CS). Transcripts were studied intently to identify recurrent themes and ideas. Data were then coded with related codes being grouped into themes and sub-themes. Repeated reflection of the codes and themes continued until all themes were clearly defined. Refinement of the definitions was completed through discussion with a second researcher (KL). The codebook can be found in Table 1.

**Table 1:** Codebook: The initial codebook was initially developed utilizing codes in line with the NASSS framework and semi-structured interview guides. Codes were subsequently discussed, refined, and finalized. All transcripts were subsequently coded

*Artificial intelligence*	
AI as an assessment tool	Use of AI to assess surgical performance and skills
AI as an educational tool	Use of AI for surgical education
AI in the operating theatre	Uses of AI within the operating theatre e.g. intraoperative navigation tools
AI outside the operating theatre	Uses of AI in the diagnosis and management of patients e.g. risk prediction models
Analytic potential	The potential of AI to provide novel insights into surgical performance and patient care
Hesitancy around AI	Hesitancy towards the use of AI in surgery or medicine in general
Liability (AI)	Responsibility for outcomes when using AI applications
Need for extensive validation and evaluation	The requirement for AI to be extensively validated before it can be used in clinical practice
Resistance to use of AI	Resistance to the implementation of AI across medicine
Videos as data	The use of videos as data for analysis and the development of digital surgical technologies
*Data protection and regulations*	
Consent	Obtaining consent from patients to record their procedure
Data protection	Protection of personal data under the UK GDPR regulations
Data ownership	Issues pertaining to ownership of the data, in terms of intellectual property
Importance of regulations	The critical role of having universal guidelines and regulations for the successful implementation of surgical video recording
Privacy	The privacy and confidentiality of patients and theatre staff when recording operative videos
*Infrastructure*	
Cybersecurity	The security of videos within digital systems
Storage	The storage of surgical procedure videos
Technical issues	Issues relating to using the equipment in the operating theatre
*The patient*	
Patient access	The ability of patients to access and watch their own surgical procedure recordings
Patient curiosity	Patient’s interest to see their procedure video
Patient misinterpretation	Patient misinterpretation of events due to misunderstanding of operative events
Use for litigation	The use of videos as medicolegal documentation
*The surgeon*	
Anxiety/self-consciousness	Feelings of anxiety felt by surgeons as a result of procedure recording
Experience with recording	Any prior experience of surgical video recording
Performance analysis	The use of videos to analyse operative performance
Resistance to recording	Resistance to the implementation of surgical video recording specifically
Transparency	Idea that routine recording encourages transparency amongst surgeons, improving care
Videos as an assessment tool	The use of video to assess surgical performance and skills
Video as an educational tool	The use of video for surgical education
*The wider surgical team*	
Change needed to routine	The changes that are required to successfully implement surgical video recording routinely
Disruption to workflow	Possible interference with workflow as a direct result of surgical video recording
Education needed for use	The education required to correctly use surgical video recording technology
Resistance to change	Resistance to the implementation of any new task, not exclusively surgical video recording
Surgery as a unique field	Challenges that are unique to the field of surgery
*The organisation*	
Efficiency	The possible impact of surgical videos on operative efficiency
Initial financial costs	Costs that are incurred purchasing and installing the equipment required to record
Lack of digitisation	A lack of digital technologies in an organisation
Objective procedure documentation	A procedure video objectively shows the events of an operation without subjectivity
Quality Improvement	The use of videos to provide overall quality improvement, hence improving patient safety and outcomes
Resource	The resource available to provide the necessary equipment, training, and maintenance for the implementation of surgical video recording
*The industry*	
Commercial benefit	Any benefit to the surgical technology industry as a direct result of surgical videos
Financial motivation	Motivation to record is influenced by financial gains
Hesitancy to third-party involvement	Hesitancy towards the involvement of third parties in the processing or handling of videos, as well as the development of digital surgery technologies
Role of industry	The role of industry in the introduction of routine surgical video recording

### Ethical approval

A full ethical review was undertaken, and ethical approval was obtained through the NHS Research Ethics Service (Ref—22/SC/0259). Informed consent was obtained from all participants prior to interviews.

## Results

### Pre- and post-pilot questionnaire

The pre-pilot questionnaire was distributed to 46 residents and 26 attendings with response rates of 21.7 and 26.9%, respectively. The total number of users was reduced to 30 during the pilot. The post-pilot questionnaire was distributed to 19 residents and 11 attendings, with response rates of 42.1 and 27.2%, respectively. All respondents in the post-pilot questionnaire were keen to adopt C-SATS^©^, and 90.9% believed C-SATS^©^ had a positive impact on training. 36.4% reported using the platform weekly, and 54.5% monthly. Full results from the questionnaires can be found in the Appendix 1–3.

### C-SATS^©^ engagement metrics

**Fig. 2:**
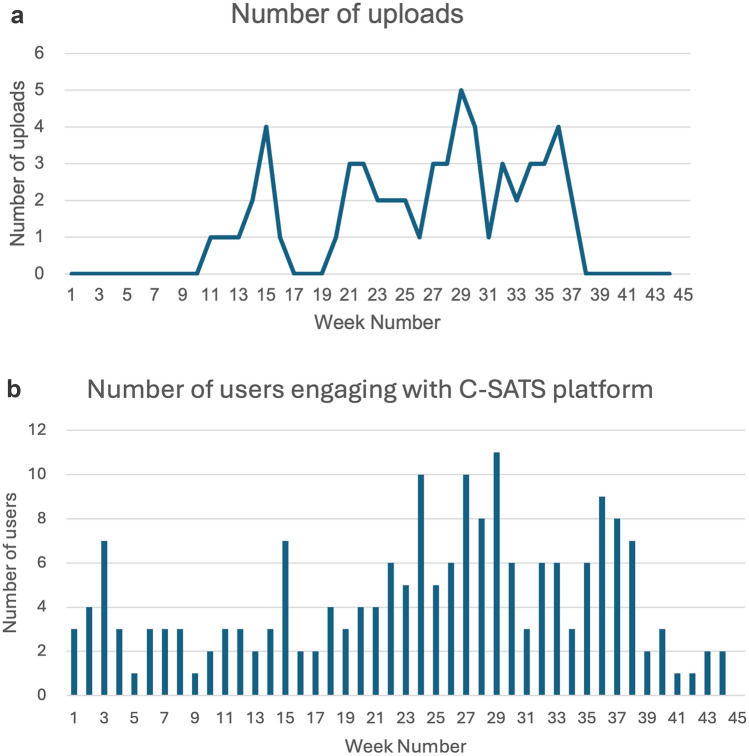
**a** Number of case uploads per week to the C-SATS© platform. A total of 57 videos were uploaded by 16 users in 24 of the 31 weeks of the pilot when the recording device was installed in operating rooms. Source: Ethicon, (Johnson and Johnson). **b** Number of users engaging with the C-SATS© platform by week. Engagement is defined as logging into the C-SATS. © platform for any purpose (*n* = 26). Purposes include accessing the video library, expert index and profile, dashboard, community dashboard, case page, academy index and video, and other page types. Source: Ethicon, (Johnson and Johnson)

Usage metrics were available from May 2022 to February 2023 (Fig. 2). Of the final 30 users, 26 accessed their accounts, and 16 uploaded videos to the platform. A total of 57 videos were uploaded in 24 of the 31 weeks in which the C-SATS^©^ device was installed in operating rooms. A consistent level of uploads failed to be achieved.

### Semi-structured interviews

#### Demographics

A total of 13 participants were interviewed: three attending surgeons, four surgical residents, three patients, one scrub nurse, one lawyer, and one industry representative. Participants were 53.8% female, and 46.2% male. Of the seven surgeons, four were involved in the C-SATS^©^ pilot (two residents and two attendings). One patient participant had previously undergone a laparoscopic procedure, with the others awaiting surgery. Thematic saturation was achieved, with no further codes created after the ninth interview.

#### Main findings

**Fig. 3:**
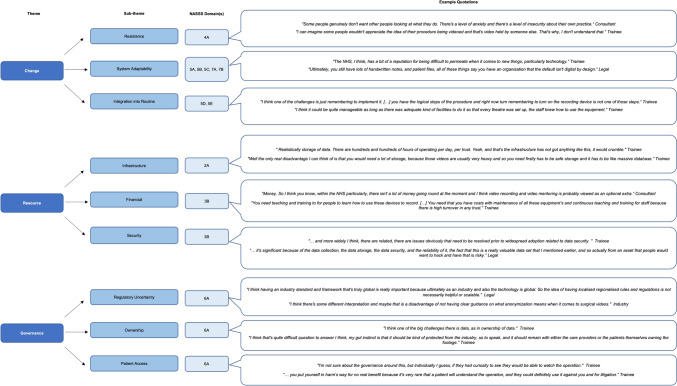
Themes and sub-themes identified in the thematic analysis relating to the barriers to the implementation of routine surgical video recording with example quotations. Each sub-theme is matched to its corresponding NASSS framework domain(s). NASSS Domains: 2A—What are the key features of the technology? 3B—What is the desirability, efficacy, safety, and cost-effectiveness (demand-side value)? 4A—What changes in staff roles, practices, and identities are implied? 5A—What is the organization’s capacity to innovate? 5B—How ready is the organization for this technology-supported change? 5C—How easy will the adoption and funding decision be? 5D—What changes will be needed in team interactions and routines? 5E—What work is involved in implementation and who will do it? 6A—What is the political, economic, regulatory, professional, and sociocultural context for program rollout? 7A—How much scope is there for adapting and coevolving the technology and the service over time? 7B—How resilient is the organization to handling critical events and adapting to unforeseen circumstances?

Three major sections were identified—value, barriers, and artificial intelligence. Within the ‘Barriers’ section, three major themes were identified, with nine sub-themes (Fig. 3).

#### Experience of recording

All professional participants had encountered surgical video recording. One attending recorded all cases. All patient participants were aware that procedures could be recorded. Of the participants involved in the C-SATS^©^ pilot, all had utilized the platform at least once. 3/7 medical professional participants had experience of consenting patients for video recording during surgery. All participants felt it was important that patients were consented explicitly for recording.

#### Value of video recording

##### Educational and quality improvement

All participants saw videos as having an educational value to the surgeons, regardless of training stage. Most participants felt the value of recording was in the ability to reflect on performance outside of the operating room where the focus can be solely on education.

##### As a record

Several participants believed video is a better form of documentation than current methods. Most saw the video as a medicolegal record, believing video to be objective and factual evidence of the procedure. However, one attending disagreed, citing a perceived inability to establish culpability from a video. One patient stated they would have been interested in accessing the video to understand the events of their procedure, in which a serious complication had occurred.

##### Commercial

Most participants agreed that videos are a valuable commercial asset, which could contribute to improvements in surgical outcomes through analysis of surgical data. A commonly suggested idea was analysis of instrument data, leading to improvements in device functionality. However, some believed industry involvement was driven by financial motivation and may lose sight of patient outcomes. There was also an awareness of a general public hesitancy towards third party involvement in handling sensitive healthcare data.

### Barriers

#### Change

##### Resistance

All medical professional participants expected there would be resistance around the introduction of routine surgical video recording. These concerns included exposure of performance at a lower competency than expected or fear of judgement from peers. Resident participants stated that recording may negatively impact performance due to the perception of being observed. However, it was acknowledged that this would be overcome if recording became standard.

##### System adaptability

All medical professionals anticipated systemic issues with adoption. Three medical professional participants believed hospitals may be limited in their abilities to adopt new technologies. Resident surgeons felt it may be challenging to demonstrate the benefit of investing in recording technology.

##### Integration into routine

All medical professional participants believed that a key challenge lies in embedding recording into the preoperative routine. This was particularly evident amongst the C-SATS^©^ pilot participants who reported that use of the device was often overlooked despite availability. Participants stated that recording should simple to employ and requires engagement from the wider surgical team. All medical professional participants suggested that inclusion of recording within the preoperative checklist would enhance implementation.

#### Resource

##### Digital infrastructure

Storage of recordings was cited as a major barrier to implementation of routine recording by all professional participants. Concerns were centered around the need for extensive storage capacity if every procedure was recorded. Several medical professional participants felt that whilst cloud-based storage is the most viable option, this creates further questions around compliance with GDPR.

##### Financial

Cost was also discussed as a major barrier, with 75% of participants believing that storage would be the biggest cost. Other identified costs included training requirements and energy-related costs. Attending participants stated hospitals may not be willing to fund recording devices due to the challenges of demonstrating a discernable and immediate benefit to the patient.

##### Security

All participants expressed the need for highly secure software systems to store surgical videos. Patients highlighted the concern felt towards the prospect of their highly sensitive surgical data being released. The legal representative also raised the issue of the vulnerability of hospitals to cyberattacks, as well as the increased risk of major data breaches if large, centralized databases are used to store surgical video.

#### Governance

##### Regulatory uncertainty

Unclear regulations with reference to anonymity in a surgical procedure recording were highlighted by both the legal and industry representative. The uncertainty was focussed on the risk of reidentification of a patient through additional data linked to the video footage, such as hospital location, or the date and time of the procedure. All patient and surgeon participants, however, perceived intraabdominal footage as anonymous.

Another identified challenge was the global variation in data protection regulations, notably between the USA and UK. Both the legal and industry representative considered the obligations in the USA to be less significant than in the UK, such that the label of anonymity is given with less weight in the USA compared to the UK. Both felt it was important to have a global framework for the labelling and handling of procedure videos, due to the global nature of the technology.

##### Ownership

Data ownership was a less eminent issue, often requiring prompting to extract views. 12/13 participants supported a hospital ownership model. One attending believed ownership should belong to the patient. However, all patients believed there was little use owning the rights to the video. All residents believed recordings should fall under the same ownership principles as other healthcare data, like imaging.

##### Patient access

Patient access to their own procedure recordings proved to be an area of uncertainty. All but one participant was in favour of patient access to recordings. One commonly cited reason for patient access was the use of video to assist in patient understanding of their condition. All medical professional participants felt there was some danger of misinterpretation of intraoperative events which could lead to increased rates of litigation.

#### Artificial intelligence

##### Role of artificial intelligence

All participants were unopposed to the use of videos for algorithm generation and believed AI has a role in the future of surgery. The belief that it would reduce surgeon workload was a recurrent theme amongst all participants.

##### Challenges

Some participants were not confident in the ability to AI to assess technical skills, whereas others felt extensive validation would be needed before its use as an assessment tool. Resident participants felt there could be challenges with the inability of AI to appreciate the intricacies of a surgical procedure, and the need to deviate from the set course, resulting in poor performance scores. All attending participants perceived a resistance amongst colleagues to the use of decision-making or intraoperative navigation tools, which may be perceived as questioning their own decision-making.

## Discussion

This study is the first to include data from a real-world recording platform, as well as utilize interviews to investigate attitudes towards routine surgical video recording and identify barriers to its adoption [12, 19, 20]. All medical professional participants in this study believed that provision of recording technology would facilitate adoption. However, only 16/30 within the C-SATS^©^ pilot used the recording device demonstrating implementation extends beyond technological availability alone. Resistance from surgeons was identified as a significant barrier in this study in line with existing evidence [8, 20]. Many believed this to be centered on concerns that recording may alter their practice, formally known as the Hawthorne effect. However, it may be argued that this is likely to be a positive change. Video provides an objective measure of various quality control measures, such as achieving the Critical View of Safety in laparoscopic cholecystectomies, definitively showing this stage has been achieved. It is not unlikely that the resistance is linked to litigation concerns. Two participants were concerned that an increase in medicolegal action would occur due to misinterpretation of events in the video by patients although this was viewed by these participants as an area to address rather than a barrier to this technology [21]. There is a fear that use of routine recording will lead to an onslaught of litigation cases [19] despite little evidence to support these claims. Participants in fact viewed videos as a positive factor in the case of litigation as they provide a better form of medicolegal evidence than the operative note.

A key theme emerging from this study was the need for recording to be integrated into the surgical workflow. However, integrating new steps within a well-established workflow is not without difficulty and lessons can be learnt with the establishment of the World Health Organization Surgical Safety Checklist [22–24]. Successful implementation will require structured education and detailed explanation of the rationale. Surgical video recording may ultimately fall under the responsibility of the wider surgical team and unless buy-in is ensured by the entire team, recording may be viewed as an unnecessary task with little immediate benefit.

The most frequently identified barrier in this study was developing the required infrastructure and its associated costs. In an ecosystem where multiple digital surgical technologies are on the market, hospital managers are under pressure to justify the costs associated with implementing surgical video recording. Current commercially available recording platforms are largely focussed on education and therefore business cases will require a growing evidence base that video-based review improves surgical performance and ultimately patient outcomes [6, 7]. A potential method for hospitals to mitigate these costs is working in conjunction with industry. This study identified the importance of videos to members of the surgical technology industry. While all participants were willing to share video data with industry, this may not be representative of the entire population. Historically, the public has been resistant to third-party involvement in healthcare with a multinational survey highlighting an aversion to the sharing of public health data with private companies [25]. Clear guidelines surrounding secondary data sharing agreements and video ownership must therefore be established to ensure responsible use of personal data for commercial purposes.

Another major theme identified surrounded experiences of consenting patients for surgical video recording. While participants found patients were generally happy to allow video recording, it is unclear where additional clauses detailing sharing with third parties would be met with similar approval. Asking patients to donate their personal health information to a company who may utilize this data for commercial gain raises potential ethical questions [26]. Standardization of processes by which patients are consented for surgical video recording and the possible downstream uses will be central to maintaining transparency with patients and retaining their trust.

Finally, anonymization of video was a common theme. Whilst surgeon and patient participants considered endoscopic recordings to be fully anonymous, both the legal and industry representatives believed the risk of identification was too high to class intracorporeal videos as anonymous. Of note, Rocher et al., described an algorithm which identified 99.98% of Americans from deidentified, incomplete databases [27]. Whilst anonymity may be important to industry in terms of allowing access, it is questionable whether detaching the video from the patient’s medical record, and thus preventing linkage of video data with clinical outcomes is useful. AI tools for decision support and risk prediction will likely require full access to the entire patient profile to reach maximum analytic potential [28–30] and therefore efforts may be better guided to improving pseudonymization of surgical procedure recordings and ensuring adequate data protection measures are in place [31].

**Fig. 4:**
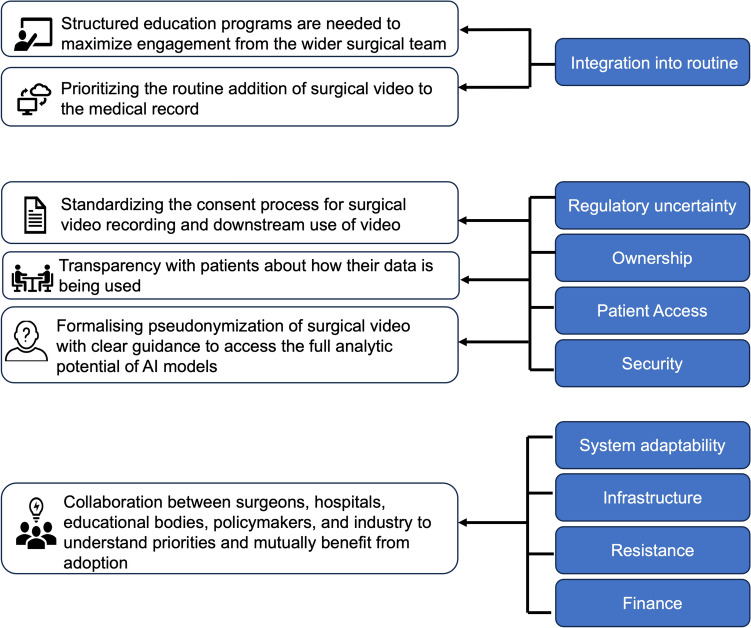
A set of recommendations aimed at surgeons, researchers, hospitals, policymakers and industry for the implementation of routine surgical video recording derived from themes obtained within this study

Through the analysis of the themes derived from these interviews, we have produced a set of recommendations for the successful implementation of routine surgical video recording (Fig. 4). These recommendations have been designed to facilitate the broad spectrum of stakeholders across surgical video recording.

This study is ultimately limited by the range of participants included within the study. Inclusion of a larger number of industry representatives would likely improve the breadth of views within our study and broader exploration through a focussed study on patient views alone around routine surgical video recording will likely enhance our understanding around failure in adoption. Furthermore, an international sample of participants would provide greater insights into the global applicability of the identified barriers. Future work may therefore consist of further semi-structured interviews or a consensus technique, such as a Delphi Consensus. It can also be argued that participants taking part in the study were more likely to be in favour of surgical video recording potentially biasing the results obtained.

## Conclusions

Barriers to implementation of routine surgical video recording exist beyond the technological level alone. This study is the first thorough exploration of these barriers utilizing semi-structured interviews. We provide recommendations for improved implementation of routine surgical video recording through the synthesis of themes derived from these interviews. Integration of video recording within the surgical workflow will be a priority and will require buy-in from the wider surgical team. Furthermore, all stakeholders must be consulted including patients, who must be engaged and educated in how their data is being used.

## Supplementary Information

Below is the link to the electronic supplementary material.

Supplementary file1 (DOCX 223 KB)
