# Supplementary material for: Barriers to the adoption of routine surgical video recording: a mixed-methods qualitative study of a real-world implementation of a video recording platform
Source: Surg Endosc. 2024 Aug 15;38(10):5793–802. doi: 10.1007/s00464-024-11174-2 (PMC11458650; doi:10.1007/s00464-024-11174-2)
Supplement: Supplementary file 1 — Supplementary file1 (DOCX 223 KB) [file 464_2024_11174_MOESM1_ESM.docx]

**SUPPLEMENTARY MATERIAL**

**APPENDIX 1 – Trainer (Attending) Pre-pilot questionnaire**

| Gender | Male | | Female | |
| --- | --- | --- | --- | --- |
|  | 100 (7) | | 0 | |
| Age (years) | **43** | **45** | **46** | **53** |
|  | 14.3 (1) | 14.3 (1) | 57.1 (4) | 14.3 (1) |
| Number of years as a attending | **5** | **7** | **9** | **18** |
|  | 14.3 (1) | 28.6 (2) | 28.6 (2) | 28.6 (2) |
| Number of residents you are supervising | **3** | **4** | **5** | **6** |
|  | 14.3 (1) | 28.6 (2) | 28.6 (2) | 28.6 (2) |
| Are you performing robotic surgery? | **Yes** | | **No** | |
|  | 57.1 (4) | | 42.9 (3) | |
| If you are not performing robotic surgery, do you plan to? | **Yes** | | **No** | |
|  | 80 (4) | | 20 (1) | |

| Question  % (n) | Strongly agree | Agree | Somewhat agree | Neither agree nor disagree | Somewhat disagree | Disagree | Strongly disagree |
| --- | --- | --- | --- | --- | --- | --- | --- |
| I am satisfied with the way I give feedback for resident procedures. | 0 | 42.9 (3) | 14.3 (1) | 14.3 (1) | 14.3 (1) | 14.3 (1) | 0 |
| I complete PBAs with my resident present. | 14.3 (1) | 28.6 (2) | 14.3 (1) | 14.3 (1) | 14.3 (1) | 14.3 (1) | 0 |
| PBAs are completed in the immediate postoperative period. | 0 | 14.3 (1) | 28.6 (2) | 28.6 (2) | 14.3 (1) | 0 | 14.3 (1) |
| PBAs completed at a delayed interval after the procedure are as reliable as those completed contemporaneously. | 0 | 42.9 (3) | 28.6 (2) | 14.3 (1) | 0 | 14.3 (1) | 0 |
| The PBA form process enables me to accurately assess my resident’s ability. | 0 | 28.6 (2) | 57.1 (4) | 14.3 (1) | 0 | 0 | 0 |
| I use my residents' past PBAs to plan their training for future lists. | 0 | 42.9 (3) | 28.6 (2) | 14.3 (1) | 0 | 14.3 (1) | 0 |
| I currently record my operative cases and am able to review and learn from them. | 14.3 (1) | 28.6 (2) | 28.6 (2) | 14.3 (1) | 0 | 14.3 (1) | 0 |
| Recording my training cases and being able to review individual steps would help me to understand my resident's learning needs. | 28.6 (2) | 57.1 (4) | 0 | 14.3 (1) | 0 | 0 | 0 |
| Reviewing operative steps that my resident found challenging using a video replay would improve feedback and training. | 42.9 (3) | 42.9 (3) | 14.3 (1) | 0 | 0 | 0 | 0 |
| Having video evidence of operative competence would more accurately assess resident's surgical ability. | 42.9 (3) | 42.9 (3) | 0 | 14.3 (1) | 0 | 0 | 0 |
| I currently have access to and routinely watch operative videos to prepare for my training cases. | 0 | 42.9 (3) | 28.6 (2) | 14.3 (1) | 0 | 0 | 14.3 (1) |
| Having a freely available library of expert cases broken down into operative steps would help prepare my residents for their cases. | 57.1 (4) | 42.9 (3) | 0 | 0 | 0 | 0 | 0 |
| Receiving objective, anonymous feedback from an expert reviewer regarding operative technique would improve learning for my residents. | 42.9 (3) | 28.6 (2) | 0 | 14.3 (1) | 14.3 (1) | 0 | 0 |
| I feel that autonomous rating and scoring of technical skills can be beneficial to my residents. | 28.6 (2) | 28.6 (2) | 14.3 (1) | 0 | 28.6 (2) | 0 | 0 |
| I am keen to adopt new digital technology in theatre. | 57.1 (4) | 14.3 (1) | 14.3 (1) | 14.3 (1) | 0 | 0 |  |
| When a new technology is introduced into the theatre it has a positive impact on training. | 42.9 (3) | 14.3 (1) | 14.3 (1) | 14.3 (1) | 14.3 (1) | 0 |  |
| How do you feel about having your training case data stored on a cloud? | 0 | 42.9 (3) | 14.3 (1) | 42.9 (3) | 0 | 0 |  |
| How do you feel about having your videos open for review by other users? | 14.3 (1) | 42.9 (3) | 14.3 (1) | 14.3 (1) | 14.3 (1) | 0 |  |
| Data security is very important to me. | 71.4 (5) | 28.6 (2) | 0 | 0 | 0 | 0 |  |
| Data ownership of my own video case is very important to me. | 42.9 (3) | 28.6 (2) | 14.3 (1) | 14.3 (1) | 0 | 0 |  |
| I would be happy for my video case data to be used to create algorithms that will be used by commercial third parties. | 28.6 (2) | 28.6 (2) | 14.3 (1) | 0 | 14.3 (1) | 0 |  |

| Question % (n) | Recording training cases | Reviewing operative steps | Video evidence of competency | Monitoring progress of technical skills | Other |
| --- | --- | --- | --- | --- | --- |
| Please select what you feel is the most important feature of C-SATs to you: | 42.9 (3) | 28.6 (2) | 14.3 (1) | 14.3 (1) |  |
|  | Yes | No |  |  |  |
| Do you have any concerns regarding autonomous rating and scoring of technical skills for your residents? | 42.9 (3) | 57.1 (4) |  |  |  |
| If you have answered yes above, please specify what concerns you may have regarding autonomous rating and scoring of technical skills. | Trainers need to be validated and UK based, this should be transparent | The need more generic feedback. | No justification |  |  |
|  | Yes | No |  |  |  |
| I currently use a digital platform to record my cases and review them. | 57.1 (4) | 42.9 (3) |  |  |  |
| Which platform? | C-SATS | | | | |
|  | Objective feedback | Learning through personal/peer videos | Quantitative feedback | Surgeon benchmarking |  |
| What do you see as the greatest benefit of video-based assessment? | 28.6 (2) | 42.9 (3) | 14.3 (1) | 14.3 (1) |  |

**APPENDIX 2 – Pre-Pilot Resident Responses**

| Gender | Male | | | | | | | | Female | | | | | | |
| --- | --- | --- | --- | --- | --- | --- | --- | --- | --- | --- | --- | --- | --- | --- | --- |
|  | 60 (6) | | | | | | | | 40 (4) | | | | | | |
| Age (years) | 29 | 34 | | | 35 | | 36 | | | 37 | | 38 | | 46 | 47 |
|  | 20 (2) | 10 (1) | | | 20 (2) | | 10 (1) | | | 10 (1) | | 10 (1) | | 10 (1) | 10 (1) |
| Current Level | **CT2/ST2**  20 (2) | | **ST3**  10 (1) | | | **ST4**  10 (1) | | **ST6**  20 (2) | | | **ST8**  20 (2) | | | **Clinical Fellow**  20 (2) | |
|  |  |  |  |  |  |  |  |  |  |  |  |  |  |  |  |
| Number of laparoscopic cases performed. | **5-20** | | | **20-50** | | | | | **50-200** | | | | **200+** | | |
|  | 10 (1) | | | 60 (6) | | | | | 20 (2) | | | | 10 (1) | | |
| Are you performing robotic surgery? | **Yes** | | | | | | | | **No** | | | | | | |
|  | 22.2 (2) | | | | | | | | 77.8 (7) | | | | | | |

|  | | | | | | | |
| --- | --- | --- | --- | --- | --- | --- | --- |
|  | Strongly agree | Agree | Somewhat agree | Neither agree nor disagree | Somewhat disagree | Disagree | Strongly disagree |
| The feedback I receive from the current PBA format enables me to understand my learning needs. | 10 (1) | 40 (4) | 40 (4) | 0 | 10 (1) | 0 | 0 |
| The feedback I receive from a PBA accurately assesses my surgical ability. | 0 | 30 (3) | 50 (5) | 10 (1) | 0 | 10 (1) | 0 |
| Reviewing a recorded PBA at a delayed interval after the operation enables me to identify opportunities for development. | 10 (1) | 40 (4) | 30 (3) | 10 (1) | 0 | 10 (1) | 0 |
| I use my past PBAs to plan my training for future lists. | 40 (4) | 40 (4) | 10 (1) | 0 | 0 | 10 (1) |  |
| I am satisfied with the way I receive feedback on my minimally invasive surgical technique. | 20 (2) | 30 (3) | 30 (3) | 10 (1) | 0 | 0 | 10 (1) |
| Recording my training cases and being able to review individual steps would help me to understand my learning needs. (10) | 30 (3) | 50 (5) | 20 (2) | 0 | 0 | 0 | 0 |
| Reviewing operative steps that I found challenging with my trainer using a video replay would improve feedback and training. (10) | 40 (4) | 30 (3) | 10 (1) | 10 (1) | 10 (1) | 0 | 0 |
| Having video evidence of operative competence would more accurately assess my surgical ability.(10) | 40 (4) | 30 (3) | 10 (1) | 20 (2) | 0 | 0 | 0 |
| Having a freely available library of expert cases broken down into operative steps would help to prepare for my training cases. (10) | 30 (3) | 40 (4) | 20 (2) | 10 (1) | 0 | 0 | 0 |
| I feel that receiving feedback on my technical skills from randomised and anonymised expert reviewers would be beneficial to my training. (0) | 20 (2) | 60 (6) | 10 (1) | 10 (1) | 0 | 0 | 0 |
| I feel that autonomous rating and scoring of technical skills can be beneficial to my training.(9) | 22.2 (2) | 33.3 (3) | 11.1 (1) | 11.1 (1) | 11.1 (1) | 11.1 (1) | 0 |
| How do you feel about having your training case data stored on a cloud? (9) | 11.1 (1) | 22.2 (2) | 11.1 (1) | 44.4 (4) | 11.1 (1) | 0 | 0 |
| How do you feel about having your training case videos open for review by other users? (9) | 11.1 (1) | 33.3 (3) | 11.1 (1) | 22.2 (2) | 11.1 (1) | 11.1 (1) | 0 |
| Data security is very important to me. (9) | 77.8 (8) | 0 | 0 | 0 | 0 | 11.1 (1) | 11.1 (1) |
| Data ownership of my own training cases is very important to me (8) | 25 (2) | 62.5 (5) | 0 | 12.5 (1) | 0 | 0 | 0 |
| I would be happy for my training case data to be used to create algorithms that will be used by a commercial third party. (9) | 22.2 (2) | 44.4 (4) | 11.1 (1) | 0 | 0 | 0 | 22.2 (2) |

|  | | | | | | | | | | | | |
| --- | --- | --- | --- | --- | --- | --- | --- | --- | --- | --- | --- | --- |
|  | Yes | No | | Strongly agree | | | |  | |  | | |
| I currently use a digital platform to record my cases and review them. (10) | 20 (2) | 70 (7) | | 10 (1) | | | |  | |  | | |
| If you answered yes above, please specify which digitial platform you use to record cases and review them. (2) | DS1, CSATS |  | |  | | | |  | |  | | |
|  | Objective feedback | Quantitative feedback | | Learning through personal and peer videos | | | | Surgeon benchmarking. | |  | | |
| What do you see as the greatest benefit of video-based assessment? (9) | 33.3 (3) | 33.3 (3) | | 33.33 (3) | | | | 0 | |  | | |
|  | Recording training cases. | Reviewing operative steps | | Video evidence of competency | | | | Monitoring progress of technical skills | | Other | | |
| Please select what you feel is the most important feature of C-SATS to you. (9) | 33.3 (3) | 11.1 (1) | | 11.1 (1) | | | | 44.4 (4) | | Video Recording and Feedback | | |
|  | Yes | No | |  | | | |  | |  | | |
| Do you have any concerns regarding autonomous rating and scoring of technical skills? (9) | 66.7 (6) | 33.3 (3) | |  | | | |  | |  | | |
| If you selected yes, please expand on your concerns: | - May not reflect true performance - Feeling judged, unsure if they will be able to scale feedback with progression and current skills level. | | | | | | | | | | | |
|  | | | Always | | Sometimes | Not Sure | Rarely | | Never | | Disagree |  |
| PBAs are completed with my trainer present. | | | 0 | | 80 (2) | 0 | 10 (1) | | 0 | | 10 (1) |  |
| PBAs are completed in the immediate postoperative period (same day). | | | 0 | | 40 (4) | 10 (1) | 20 (2) | | 20 (2) | | 10 (1) |  |
| PBAs completed at a delayed interval after the procedure are as reliable as those completed contemporaneously. | | | 0 | | 40 (4) | 40 (4) | 10 (1) | | 0 | | 10 (1) |  |
| I currently record my operative cases and I am able to review them and learn from them. (10) | | | 30 (3) | | 20 (2) | 0 | 30 (3) | | 10 (1) | | 10 (1) |  |
| I currently have access to and routinely watch operative videos to prepare for my training cases. (10) | | | 30 (3) | | 40 (4) | 0 | 20 (2) | | 0 | | 10 (1) |  |
|  | | | 0 | | 0-1 | 1-2 | 2-5 | | 5-10 | | >10 | Strongly agree |
| How many trainers assess your technical competency in your job? | | | 0 | | 10 (1) | 20 (2) | 60 (6) | | 0 | |  | 10 (1) |

**APPENDIX 3 – Post pilot questionnaire responses**

| *Appendix 3c: post-pilot questionnaire responses (cont.)* | | | | | | | | | | | | |
| --- | --- | --- | --- | --- | --- | --- | --- | --- | --- | --- | --- | --- |
| % (n) |  | | | | | |  | | | | | |
| Gender | Male | | | | | | Female | | | | | |
|  | 72.7 (8) | | | | | | 27.3 (3) | | | | | |
| Age | 27 | 29 | | 34 | | 35 | 37 | 39 | | 46 | | 47 |
|  | 9.1 (1) | 9.1 (1) | | 18.2 (2) | | 18.2 (2) | 9.1 (1) | 9.1 (1) | | 18.2 (2) | | 9.1 (1) |
| What is your specialty? | Colorectal | | UGI | | Gynaecology | | Urology | | HPB | | Bariatric | |
|  | 45.5 (5) | | 18.2 (2) | | 9.1 (1) | | 18.2 (2) | | 9.1 (1) | | 0 | |
| What is your grade? | CT1-CT2 | | ST3 | | ST4 | | ST8 | | Post CCT | | Attending | |
|  | 18.2 (2) | | 9.1 (1) | | 18.2 (2) | | 9.1 (1) | | 18.2 (2) | | 27.3 (3) | |

|  | Daily | Weekly | Monthly | Never |  |  |  |
| --- | --- | --- | --- | --- | --- | --- | --- |
| How often did you use CSATS? | 0 | 36.4 (4) | 54.5 (6) | 9.1 (1) |  |  |  |
| % (n) | Strongly agree | Agree | Somewhat agree | Neither agree nor disagree | Somewhat disagree | Disagree | Strongly Disagree |
| I believe C-SATS enhanced my training and feedback that I already receive | 18.2 (2) | 45.5 (5) | 18.2 (2) | 18.2 (2) | 0 | 0 | 0 |
| The C-SATS feedback I receive is equally or more valuable to PBAs | 18.2 (2) | 54.5 (6) | 18.2 (2) | 9.1 (1) | 0 | 0 | 0 |
| I like being able to access my videos and watch them back on C-SATS | 63.6 (7) | 27.3 (3) | 0 | 9.1 (1) | 0 | 0 | 0 |
| Recording cases on C-SATS in the OR was easy | 18.2 (2) | 27.3 (3) | 27.3 (3) | 9.1 (1) | 18.2 (2) | 0 | 0 |
| The C-SATS library of videos is very varied and has been helpful in planning my future cases. | 27.3 (3) | 36.4 (4) | 27.3 (3) | 9.1 (1) | 0 | 0 | 0 |
| Watching my videos and C-SATS case reports helped me to plan for future cases. | 27.3 (3) | 36.4 (4) | 18.2 (2) | 18.2 (2) | 0 | 0 | 0 |
| The C-SATS feedback process accurately assess resident's ability. | 9.1 (1) | 18.2 (2) | 54.5 (6) | 18.2 (2) | 0 | 0 | 0 |
| Recording my training cases and being able to review individual steps has helped me to understand my resident's learning needs. | 18.2 (2) | 54.5 (6) | 9.1 (1) | 18.2 (2) | 0 | 0 | 0 |
| Reviewing operative steps which resident found challenging using a video replay has improved feedback and training. | 27.3 (3) | 63.6 (7) | 0 | 9.1 (1) | 0 | 0 | 0 |
| Having video evidence of operative competence has allowed for more accurate assessment of residents' surgical ability. | 18.2 (2) | 54.5 (6) | 18.2 (2) | 9.1 (1) | 0 | 0 | 0 |
| I currently have access to and routinely watch operative videos to prepare for my training cases. | 18.2 (2) | 27.3 (3) | 45.5 (5) | 9.1 (1) | 0 | 0 | 0 |
| Having a freely available library of expert cases broken down into operative steps has helped to prepare my residents for their cases. | 18.2 (2) | 45.5 (5) | 36.4 (4) | 0 | 0 | 0 | 0 |
| Receiving objective, anonymous feedback from an expert reviewer regarding operative technique has positive impact on learning for my residents. | 27.3 (3) | 45.5 (5) | 27.3 (3) | 0 | 0 | 0 | 0 |
| I am keen to adopt C-SATS in theatre | 45.5 (5) | 45.5 (5) | 9.1 (1) | 0 | 0 | 0 | 0 |
| I feel that introducing C-SATS into the theatre had a positive impact on training. | 36.4 (4) | 45.5 (5) | 9.1 (1) | 9.1 (1) | 0 | 0 | 0 |
| How did you feel about your feedback received from C-SATS on your videos by other users? | 9.1 (1) | 45.5 (5) | 36.4 (4) | 9.1 (1) | 0 | 0 | 0 |
| My data was secure and anonymised on C-SATS | 18.2 (2) | 63.6 (7) | 9.1 (1) | 9.1 (1) | 0 | 0 | 0 |
| I had ownership of my videos and data uploaded on C-SATS platform. | 18.2 (2) | 0 | 27.3 (3) | 36.4 (4) | 9.1 (1) | 9.1 (1) | 0 |
| I would be happy for my video case data to be used to create algorithms that will be used by commercial third parties. | 0 | 36.4 (4) | 27.3 (3) | 9.1 (1) | 0 | 27.3 (3) | 0 |

|  | | | | | | | | | |
| --- | --- | --- | --- | --- | --- | --- | --- | --- | --- |
| % (n) | Ability to record and upload cases | Receive and expert case review | Submit comments or questions to expert reviewer | Watching case videos | Participating in telementoring | Watching high scoring videos where you are not the performing surgeon | | I did not find it valuable |  |
| Rate the aspects of C-SATS you found most helpful to your practice: | 45.5 (5) | 18.2 (2) | 0 | 18.2 (2) | 9.1 (1) | 0 | | 9.1 (1) |  |
|  | Quantitative technical skills | Qualitative comments from community | Curated expert reviews | Surgical video capture | Expert case studies | Operative insights e.g. procedure time and steps | | Video library | I did not find it valuable |
| Which aspects of the C-SATS case report you received were most helpful to your practice: | 9.1 (1) | 18.2 (2) | 9.1 (1) | 27.3 (3) | 18.2 (2) | 9.1 (1) | | 9.1 (1) | 0 |
|  | Recording training cases | Reviewing operative steps | Video evidence of competency | Monitoring progress of technical skills | Other | |  | | |
| Please select what you feel the most important features of C-SATS to you: | 36.4 (4) | 36.4 (4) | 9.1 (1) | 18.2 (2) | 0 | |  | | |
| If you selected other, please specify what you feel is the most important feature of C-SATS to you: | - Video library has been most helpful - Connectivity and peer support - It needs to be validated against current gold standard for assessing technical competency in the UK training system. | | | | | | | | |
|  | Yes | No |  |  |  | |  | | |
| Do you have concerns regarding autonomous rating and scoring of technical skills for your residents | 36.4 (4) | 63.6 (7) |  |  |  | |  | | |
| What do you think are the limitations of C-SATS? | - It needs to be validated as an educational tool and benchmarked against the current standards for UK training. - I feel uncomfortable broadcasting my cases to everyone. Privacy and limited access can help with that - It does not account for factors other than surgeon which can also influence outcomes - Technical/kit/equipment issues that prevent its use - Needs global adoption to have impact, needs both resident and trainer buy in, needs UK validation/video trainer assessors, business model needs to be cost effective and not on a per user license model - It requires a device to upload the videos | | | | | | | | |
| What do you think could be improved on the C-SATS platform? | - Searching operations in the library by steps - You really need to improve the flexibility of logging and video recording - Transparency of what future uses of the data may be engagement with deanery to validate at scale. - Easier availability | | | | | | | | |

**APPENDIX 4: The NASSS Framework (Greenhalgh et al., 2017)**
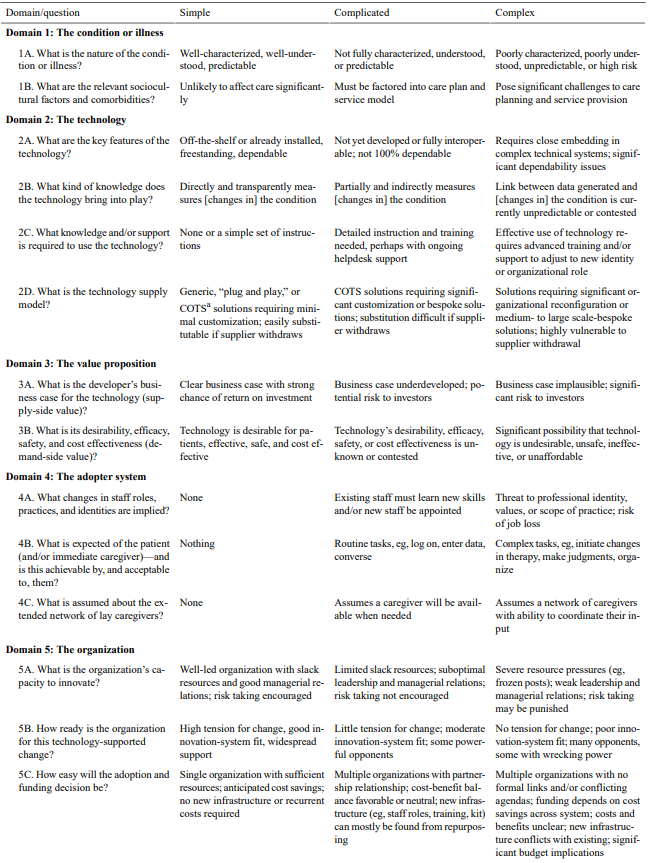


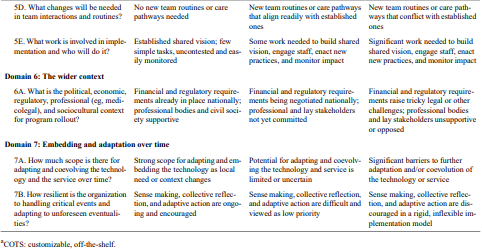


**APPENDIX 5: Topic guide for surgical staff and professional participants with NASSS Framework domains.**

|  | **Question** | **NASSS Framework Domain(s)** |
| --- | --- | --- |
| **1** | Can you start by telling me a little bit about your role? |  |
| **2** | Would you be able to share your experiences of intraoperative recording? | 1A, 1B |
| **3** | What methods of surgical video capture are you aware of? | 2A |
| **4** | What do you think is the value of video recording during surgery?   - Value to surgical team. - Value to patient. - Value to industry and commercial entities. - Are you able to share personal experience of benefit? - How do you think the value will change, if at all, over the next three-five years? | 3A, 3B  4A, 4B  5A  6A |
| **5** | What do you think are the potential disadvantages of routine video recording during surgery? | 3B |
| **6** | What are the challenges of using current methods of recording?   - How would you rate their performance? - How dependable are they? - Do staff understand what it does, and why video is being recorded? | 2B, 2C, 2D |
| **7** | How do you think routine intraoperative video recording affects your role within the surgical team?   - What is your opinion? - How do you think this might change in the next three-five years? | 2C, 2D  4A  5D |
| **8** | What is your experience of consenting patients for video recording during surgery?   - Could it be improved? If yes, how so? | 4A |
| **9** | Who do you think should own the rights to the video?   - Should patients be able to access and potentially share the video? - Should there be a difference if the video is collected for quality improvement or research purposes? | 2B  6A |
| **10** | A big concern among surgeons is how it may affect the privacy of the surgical team and especially how video may be used for purposes of litigation. What is your opinion of this? | 4A  6A |
| **11** | Beyond your own surgical team, what do you believe have been the challenges for your trust or hospital in adopting intraoperative video recording? What challenges did you face? What challenges are still present?   - Data storage - Information governance - Culture | 5A-E  6A  7A, 7B |
| **12** | What are the key challenges to the adoption of video recording routinely in the operating theatre?   - Culture   Legal challenges | 5B, 5D, 5E.  6A  7A |
| **13** | Artificial intelligence is being increasingly used in surgery. How would you feel about your videos being used by third-party companies to generate algorithms that are used in surgical AI technology?   - What do you think the role of AI in surgery is? - Do you think AI could be used in the determination of training progression?   How do you feel about this? | 2B  4A, 4B  6A  7A |
| **14** | Do you think professional organisations support or oppose the introduction of intraoperative recording?  RCS/GMC – impact on future credentialing | 6A  7A. |
| **15** | How could we learn from other areas within medicine? Or even from other industries? | 5C |
| **15** | Do you have any further questions or points to make which we may not have covered? |  |

**APPENDIX 6: Topic guide for patient participants with NASSS Framework domains**

|  | **Question** | **NASSS Framework**  **Domain** |
| --- | --- | --- |
| **1** | Can you start by telling me a bit about your experiences of having an operation? |  |
| **2** | Have you ever been offered to see pictures or videos of your procedure? | 1A, 1B |
| **3** | Are you aware of the move towards capturing videos in surgery? | 1A |
| **4** | What do you think the value of video recording during surgery?   - Value to the surgical team. - Value to the patient. - Are you able to share personal experience of benefit? - Value to industry and commercial entities. - How do you think the value will change over the next three-five years? | 3A, 3B  4A, 4B |
| **5** | What do you think are the potential disadvantages of video recording during surgery?   - Data security - Identifying individuals - Make operations longer | 2B  3B  5A, 5D.  6A |
| **6** | If your surgical team were recording your procedure, would you want to be explicitly consented for this?   - When would you want to know? - How much information would you want to know? - Would you want to know how your video could be used in the future? | 2B  4B |
| **7** | Would you be interested in owning a copy of your procedure video if you had the opportunity?  Who do you think should own the rights to the video? | 4B  6A |
| **8** | What are your main concerns if the video were to be recorded during your surgery? | 4B |
| **9** | Would you be happy for your videos to be used for the following circumstances?   - Training of doctors - In complaints or investigations - To share clinical information about your case with other medical professionals (e.g. another surgeon involved in your case) - Insurance   Development of new healthcare technology | 3A, 3B  6A |
| **10** | Do you have any further questions or points to make which we may not have covered? |  |

**APPENDIX 7: Standards for Reporting Qualitative Research (SRQR) checklist, adapted from the GoodReports.org checklist (https://www.goodreports.org/reporting-checklists/srqr/info/) O’Brien et al., 2014.**

| **No.** | **Topic** | **Page Number** |
| --- | --- | --- |
| **Title and abstract** | | |
| S1 | Title | 1 |
| S2 | Abstract | Abstract |
| **Introduction** | | |
| S3 | Problem formulation | 2-3 |
| S4 | Purpose or research question | 3 |
| **Methods** | | |
| S5 | Qualitative approach and research paradigm | 4-7 |
| S6 | Researcher characteristics and reflexivity | 4-7 |
| S7 | Context | 2-3 |
| S8 | Sampling strategy | 6 |
| S9 | Ethical issues pertaining to human subjects | 7 |
| S10 | Data collection methods | 4-7 |
| S11 | Data collection instruments and technologies | 4-7 |
| S12 | Units of study | 4-7 |
| S13 | Data processing | 6-7 |
| S14 | Data analysis | 6-7 |
| S15 | Techniques to enhance trustworthiness | 6-7 |
| **Results** | | |
| S16 | Synthesis and interpretation | 8-14 |
| S17 | Links to empirical data | 8-14 |
| **Discussion** | | |
| S18 | Integration with prior work, implications, transferability, and contributions to the field | 15-17 |
| S19 | Limitations | 17 |
| **Other** | | |
| S20 | Conflicts of interest | 18 |
| S1 | Funding | 18 |
